# Novel Nitrogen Heterocycle–Hydroxamic Acid Conjugates Demonstrating Potent Anti-Acute Lymphoblastic Leukemia Activity: Induction of Endogenous Apoptosis and G0/G1 Arrest via Regulation of Histone H3 Acetylation and AKT Phosphorylation in Jurkat Cells

**DOI:** 10.3390/cells14221822

**Published:** 2025-11-20

**Authors:** Lingjie Wu, Li Zhao, Liping Wang, Yi Lu, Gaojie Lou, Bin Zhang, Ning Wang

**Affiliations:** 1 School of Pharmacy, Health Science Center, Ningbo University, Ningbo 315211, China; 2311390166@nbu.edu.cn (L.W.); 236004797@nbu.edu.cn (Y.L.); 236002485@nbu.edu.cn (G.L.); 2 Institute of Drug Discovery Technology, Ningbo University, Ningbo 315211, China; 2111072066@nbu.edu.cn (L.Z.); 2311130047@nbu.edu.cn (L.W.)

**Keywords:** histone deacetylase, inhibitor, apoptosis, cell cycle arrest, proteomics, anti-cancer mechanism

## Abstract

Epigenetics garnered significant scientific interest in recent decades, with histone acetylation emerging as the most prevalent epigenetic deregulation process observed in malignancies. The clinical application of histone deacetylase (HDAC) inhibitors faced challenges, including complex therapeutic mechanisms and inconsistent treatment outcomes. In Acute Lymphoblastic Leukemia (ALL), the dysregulation of HDAC activity presents a promising therapeutic target. To investigate cellular-level tumor suppression by HDAC inhibitors possessing potent target engagement, we developed two novel azetidine-hydroxamic acid conjugates. Compared to N-hydroxy-4-((quinolin-4-ylamino)methyl)benzamide (NBU-1), N-hydroxy-6-((5-methyl-4-nitro-9-oxo-9,10-dihydroacridin-1-yl)amino)hexanamide (NBU-2) demonstrated enhanced inhibitory activity against HDAC1 (class I) and HDAC6 (class II) with IC_50_ values of 7.75 nM and 7.34 nM, respectively, consistent with binding mode analysis and docking energy calculations. In vitro evaluation across 12 tumor cell lines revealed NBU-2’s potent antiproliferative effects, particularly against the ALL-derived Jurkat cells (IC_50_ = 0.86 μM). Subsequent mechanistic studies were therefore conducted in this ALL model. Proteomic profiling indicated its potential involvement in modulating AKT signaling and histone modification pathways in Jurkat cells. Mechanistic investigations demonstrated that NBU-2 elevated histone acetylation while suppressing AKT phosphorylation. This compound altered apoptotic regulators by downregulating Bcl-2 and Bcl-XL expression while upregulating BAX, ultimately activating Caspase-9 and Caspase-3 to induce apoptosis. Cell cycle analysis revealed NBU-2-mediated G0/G1 arrest through reduced expression of Cyclin D1 and CDK4, diminished Rb protein phosphorylation, and increased p21 expression. These findings propose a strategic framework for developing next-generation HDAC inhibitors for ALL treatment and elucidating their mechanism-specific anti-cancer actions.

## 1. Introduction

Acute lymphoblastic leukemia occurs when there is malignant transformation and uninhibited proliferation of T-lymphoblasts in the body. Acute T-lymphoblastic leukemia (T-ALL) has been classified as a high-risk acute lymphoblastic tumor because of its poor response to conventional chemotherapy and susceptibility to relapse [1]. At this stage, investigational drugs targeting T-ALL are mainly classified into the following categories: (1) proteasome inhibitors (Bortezomib) [2]; (2) HDAC inhibitors (Vorinostat, Belinostat, Panobinostat Lactate, etc.) [3]; (3) Bcl-2 inhibitors (venetoclax) [4] and mitogen-activated protein kinase inhibitors, PARP inhibitors [5]. Among them, HDAC inhibitors are one of the important categories. Given the pivotal role of epigenetic dysregulation in T-ALL pathogenesis, HDAC inhibitors represent a particularly promising therapeutic strategy for this disease.

Histone deacetylases (HDACs) are important epigenetic regulators, and their abnormalities are closely related to malignant tumor phenotypes [6]. The existing HDAC inhibitors are classified into four categories based on their structures: carboxylic acids (valproate, butyrate), hydroxamic acids (vorinostat), cyclic peptides (romidepsin), aminobenzamides (mocetinostat) [7,8]. Nonetheless, the uniform structure of hydroxamic acids HDAC inhibitors allows them to selectively impede tumor cells at low doses and concentrations while having minimal harmful effects on normal cells.

To better understand how HDAC inhibitors fight against tumors at the cellular level and to explore their specific application in T-ALL, two novel HDAC inhibitors (NBU-1 and NBU-2) were designed and synthesized based on hydroxamic acid fragments. The structural differences between the two compounds are as follows: (1) The CAP region of NBU-1 is a bicyclic conjugated quinoline ring, while NBU-2 is a tricyclic conjugated acridine ring. Both of these two types of heterocycles are reported scaffolds with certain anti-tumor activity. In our preliminary structure-activity relationship (SAR) study [9], the 4-nitro group and 5-methyl group were introduced onto the acridone scaffold of NBU-2; (2) The types of linker chains in these two compounds are different. NBU-1 is a rigid chain (benzene ring), whereas NBU-2 is a flexible chain (containing five methylene groups). Based on in vitro target activity screening, we found that the IC_50_ values of NBU-1 against HDAC1 (Class I) and HDAC6 (Class II) are 242 and 114 nM, respectively, while the activity of NBU-2 is significantly increased, with the IC_50_ values of 7.75 and 7.34 nM, respectively. Therefore, NBU-2 is selected for further in-depth investigation of its anti-T-ALL mechanism in this study, using the Jurkat cell model. HDAC1 inhibition primarily affects nuclear histone acetylation and transcriptional reprogramming. Increased levels of H3 and H4 acetylation further upregulate tumor suppressor genes such as CDKN1A (p21) and BAX, leading to G1/S phase arrest and apoptosis [10]. Conversely, HDAC6 inhibition mainly acts on the cytoplasm, disrupting the AKT survival pathway by regulating non-histone substrates, including α-tubulin and HSP90 [11,12]. Therefore, simultaneous inhibition of both HDAC1 and HDAC6 produces a potent antiproliferative effect.

## 2. Materials and Methods

### 2.1. Chemistry

Commercial reagent-grade chemicals and solvents were used without further purification. All the water mentioned in the reactions was ultra-pure water. Thin-layer chromatography was carried out using plate silica gel F254. Silica gel for column chromatography was 100–200 mesh and 200–300 mesh. All chemical yields are unoptimized and generally represent the result of a single experiment. Melting points (mp) were recorded on an auto melting point system (WPS-1A, Shanghai Suoguang Electric Technology Co., Ltd., Shanghai, China). ^1^H NMR and ^13^C NMR spectra of the compounds were obtained using a Bruker 600 MHz (Beijing, China). Chemical shifts (δ_H_, δ_C_) and coupling constant values (J) are given in ppm and Hz, respectively. Splitting patterns are indicated as s, singlet; d, doublet; t, triplet; q, quartet signal; m, multiplet. High-Resolution Mass spectrometers (HRMS) were recorded on Waters G2-XS QTof (Shanghai, China) and Agilent Technologies 6224 TOF LC/MS system (Beijing, China). Please see Supplementary Material for the structures and hydrogen atom numbers of intermediate **3**, NBU-1, and NBU-2.

#### 2.1.1. Synthesis and Characterization of NBU-1

Step 1: Methyl 4-(aminomethyl)benzoate hydrochloride **1** (5.77 mmol) and NaOH (14.9 mmol) were stirred in anhydrous methanol (25 mL) for 1 h at 0 °C. After the reaction was completed, the mixture was partitioned between dichloromethane (30 mL) and water (30 mL). The organic layer was worked up to give a residue that was dissolved in 1,4-dioxane (30 mL) along with 4-bromoquinoline **2** (4.81 mmol), Palladium acetate (0.29 mmol), DPEPhos (0.58 mmol), and potassium phosphate (14.42 mmol). The reaction was carried out under the protection of nitrogen, and the mixture was stirred at 100 °C until TLC (petroleum ether/ethyl acetate = 1:2, *v*/*v*) indicated complete consumption of the starting material. After extraction with dichloromethane (3 × 20 mL) and saturated brine (3 × 20 mL), the organic layer was dried over anhydrous MgSO_4_ and concentrated in vacuo. Purification by column chromatography (Eluent: petroleum ether/ethyl acetate = 1:5, *v*/*v*) afforded product **3** in 68% yield. m.p.: 124.1–124.3 °C, ^1^H NMR (600 MHz, Chloroform-*d*) δ 8.49 (d, J = 5.3 Hz, H4), 8.04–8.02 (m, H10/11), 8.01 (dd, J = 8.5, 0.8 Hz, H6), 7.85 (dd, J = 8.4, 0.7 Hz, H3), 7.67–7.63 (m, H2), 7.47–7.44 (m, H1/9/12), 6.36 (d, J = 5.4 Hz, H5), 5.79 (s, H7), 4.63 (d, J = 5.3 Hz, H8), 3.91 (s, H13). ^13^C NMR (151 MHz, Chloroform-*d*) δ 166.87, 150.71, 149.70, 148.08, 142.86, 130.35, 129.81, 129.52, 127.26, 125.20, 119.59, 118.80, 99.62, 52.32, 47.29.

Step 2: Compound **3** (0.72 mmol) and hydroxylamine hydrochloride **4** (10.8 mmol) were dissolved in methanol (10 mL), then sodium tert-butoxide (1.8 mmol) was added to adjust the pH of the solution to about 11. The reaction was maintained at 0 °C for 20 min, then warmed to room temperature and stirred for 2–3 h until TLC (ethyl acetate/ethanol = 6:1, *v*/*v*) showed full conversion. Addition of 2 M HCl to pH 6–8 induced precipitation. The solid was collected by filtration, dried, and NBU-1 was obtained without further purification. White solid, yield: 73%, m.p.: 145.8–147.0 °C. ^1^H NMR (600 MHz, DMSO-*d*_6_) δ 8.32 (d, *J* = 8.3 Hz, H4), 8.28 (d, *J* = 5.3 Hz, H6), 8.05–8.03 (m, H13), 7.78 (d, *J* = 8.3 Hz, H3), 7.70 (d, *J* = 8.3 Hz, H10/11), 7.64–7.60 (m, H2), 7.48–7.41 (m, H1/9/12), 6.28 (d, *J* = 5.4 Hz, H5), 4.59 (d, *J* = 5.9 Hz, H8).^13^C NMR (151 MHz, DMSO-*d*_6_) δ 163.95, 150.56, 149.67, 148.30, 142.19, 131.66, 129.08, 128.83, 127.03, 126.75, 125.65, 124.06, 121.73, 118.94, 99.00, 45.27. HR-MS (ESI): Calcd for [M+H]^+^ 294.1243; Found: 294.1214. (See Supplementary Material for Raw spectrograms).

#### 2.1.2. Synthesis and Characterization of NBU-2

The preparation of intermediates **7**, **8**, and **10** is according to our previously published literature [9], and the final reaction step is described below. Intermediate **10** (1.0 eq) and hydroxylamine hydrochloride (15 eq) were dissolved in methanol (10 mL) in an ice bath, then sodium methylate was added to adjust the pH of the solution to about 11. The reaction was carried out at 0 °C for 20 min, then at room temperature for 2–4 h until the TLC showed the disappearance of the starting material. 2 mol/L HCl (a.q.) was added until pH = 6–8, and the precipitate was formed. NBU-2 was obtained after filtration and purified by column chromatography, eluted with ethyl acetate. Yellow solid, yield: 16.5%, m.p.: >200 °C. ^1^H NMR (600 MHz, DMSO-*d*_6_) δ 12.51 (s, H4), 11.77 (t, *J* = 5.1 Hz, H8), 10.37 (s, H15), 8.68 (s, H14), 8.34 (d, *J* = 9.8 Hz, H5), 8.05 (d, *J* = 7.9 Hz, H7), 7.68 (d, *J* = 7.0 Hz, H2), 7.32 (t, *J* = 7.6 Hz, H1), 6.58 (d, *J* = 9.9 Hz, H6), 3.44 (q, *J* = 6.6 Hz, H9), 2.53 (s, H3), 1.99 (t, *J* = 7.4 Hz, H13), 1.72–1.67 (m, H10), 1.61–1.56 (m, H12), 1.43–1.38 (m, H11). ^13^C NMR (151 MHz, DMSO-*d*_6_) δ 179.53, 169.42, 157.85, 140.12, 137.02, 134.90, 133.77, 125.87, 123.91,123.62, 122.31, 120.91, 103.96, 103.71, 42.89, 32.62, 28.54, 26.51, 25.27, 16.43; HR-MS (ESI): Calcd for [M+H]^+^ 399.1668; Found: 399.1669. (See Supplementary Material for Raw spectrograms).

### 2.2. In Vitro HDACs Inhibition Assays

Enzymatic assays were performed by Shanghai ChemPartner according to the HDAC kit protocol. Inhibition of HDAC1 and HDAC6 by NBU-1 and NBU-2 was assessed using Vorinostat (SAHA) as a positive control. Fluorescence was recorded at 355 nm excitation and 460 nm emission on a Synergy MX reader (PerkinElmer Life Sciences, Boston, MA, USA). IC_50_ values were derived from dose–response curves.

### 2.3. MTT Assay

K562, human erythroleukemia line; HL-60, human leukemic cell line; Jurkat, human leukemic T cell line; CCRF-CEM, human leukemic lymphoblasts; U937, human histiocytic lymphoma; HCT-116, human colorectal cancer cell line; A549, human lung cancer cell line; Hep G2, human hepatoma cell lines; MDA-MB-231, MCF-7, human breast cancer cell line; 4T1, mouse breast cancer cell line; PC12, pheochromocytoma of the rat adrenal medulla; HUVEC, human umbilical vein endothelial cells. These cells were purchased from the National Collection of Authenticated Cell Cultures (Shanghai, China).

The concentration of the cell suspension was adjusted to the appropriate concentration with fresh medium (the number of cells was about 30–40% of the bottom area of the well plate). 150 μL (about 1 × 10^4^ cells) of prepared cell suspension containing different concentrations of NBU-2 were added to a 96-microtiter plate, and 3 replicate wells were set up for each concentration. If the cells were adherent, the cells were first allowed to grow overnight in 96 microtiter plates until the cells were adherent, and then the old medium was replaced with the drug-containing medium. The plates were incubated in a 5% CO_2_ cell incubator at 37 °C for 72 h. Then, 15 μL of MTT solution was added to each well, 100 μL of 10% SDS solution (pH = 2) was added after 4 h, and the plate was incubated at 37 °C with 5% CO_2_ overnight to fully dissolve the crystals. Finally, the absorbance value of each well was measured at 570 and 655 nm.

### 2.4. CCK-8 Assay

The cells (about 1 × 10^4^ cells) were treated with different concentrations of NBU-2 and DMSO control for different times (24 h, 48 h, 72 h), with 3 replicate wells for each concentration. Incubate at 37 °C in a 5% CO_2_ cell culture incubator. After a predetermined time, 10 μL of CCK-8 was added to each well and incubated for 2 h. The absorbance was measured at the excitation wavelength of 450 nm.

### 2.5. Proteomics

The cell cultivation was completed in a 60 mm plate; the number of cells accounted for roughly 30–40% of the orifice. We stimulated cells with 1 μM and 5 μM of NBU-2 and 0.1% DMSO. Each concentration had a triplicate experiment. After 48 h, the cells were collected, about 200 μL of NP-40 Lysis Buffer containing phosphatase inhibitor and protease inhibitor was added, and the cells were lysed to release the proteins. Measure the protein concentration of each group of samples using the BCA protein quantification method. The disulfide bonds in the protein structure were reduced using TCEP and then alkylated with IAA. Samples were added to a centrifugal filtration unit with an ultrafiltration membrane, and proteins were digested using trypsin 2 times, the first time for 4 h and the second time overnight with the addition of an appropriate amount of trypsin. Centrifuge at 14,000× *g* for 10 min on the next day, collect the lower layer of liquid, and spin-dry to powder in a vacuum. Samples were reconstituted using 0.1% formic acid, and approximately 1 μg of protein was taken for detection using mass spectrometry [13].

Mass spectrometry analysis was performed using an Orbitrap Fusion Lumos mass spectrometer (Thermo Fisher Scientific, Shanghai, China). Chromatographic separations were performed on an Acclaim PepMap C18 column using a 60 min linear gradient elution program: the proportion of mobile phase B (acetonitrile containing 0.1% formic acid) was increased from 2% to 35%, and the flow rate was maintained at 250 nL/min. Raw data were processed using Proteome Discoverer 2.3 software (Thermo Fisher Scientific, Shanghai, China) and compared with the UniProt human protein database. Peptide-mass spectrometry matching (PSM) was filtered using the Percolator algorithm, with a false positive rate (FDR) of less than 1% [14].

### 2.6. Apoptosis Assay

Apoptosis was assessed using an Annexin V-FITC Apoptosis Detection Kit according to the manufacturer’s instructions. Briefly, cells were double-stained with fluorescein isothiocyanate (FITC)-conjugated Annexin V and propidium iodide (PI). Jurkat cells were cultured to the appropriate density. Three sets of concentration gradients, namely 0.2 μM, 1 μM, and 5 μM, were established while DMSO was used as the control. The Jurkat cells were cultured for 48 h. Centrifuge at 1000× *g* for 5 min to precipitate the cells. We collected the cells, added the staining solution according to the instructions of the Annexin V-FITC Apoptosis Detection Kit to complete the staining, and immediately tested the cells on Flow cytometry.

### 2.7. Cell Cycle and Apoptosis Assay

Jurkat cells were cultured to the appropriate density. Jurkat cells were stimulated with three concentration gradients of 0.2 µM, 1 µM, and 5 µM of NBU-2, and DMSO (as a control) for 48 h. Cells were collected, fixed overnight using pre-cooled 70% ethanol, and then stained according to the instructions. Subsequently, flow cytometry was used to detect using flow cytometry.

### 2.8. Reactive Oxygen Detection

Cells were treated with 0.2 µM, 1 µM, 5 µM, and DMSO for 48 h. Then the probes were loaded using serum-free medium and incubated for 20 min at 37 °C in a cell incubator. Next, the cells were collected. Fluorescence intensity was measured using a 488 nm excitation wavelength and 525 nm emission wavelength.

### 2.9. Western Blot

Jurkat cells were stimulated with NBU-2 (0.2 µM, 1 µM, 5 µM) and DMSO, respectively, for 48 h. Cells were lysed by the addition of appropriate RIPA Lysis Buffer (containing protease inhibitors and phosphatase inhibitors), and protein concentrations were determined using the BCA assay. Centrifuge the above cell lysates at 12,000× *g* for 10 min at 4 °C and retain the supernatant. After diluting the samples to the same concentration with lysate, add the corresponding volume of SDS-PAGE protein uploading buffer (5×), and the proteins were denatured by boiling at 100 °C for 10 min. According to the molecular weight of the target protein, we selected the corresponding concentration of the gel for electrophoresis. After electrophoresis, the proteins on the gel were transferred to the PVDF membrane. The transfer time could be adjusted appropriately according to the molecular weight of the proteins. Subsequently, the corresponding protein primary antibody was taken, and the corresponding antibody was diluted according to the instructions and incubated overnight at 4 °C on a shaker. The following day, it was conjugated to the secondary antibody. The protein bands were then visualized under the ChemiDoc system using the chemiluminescent developer. Antibodies HDAC1 (Cat#10197-1-AP), HDAC6 (Cat#12834-1-AP), Bax (Cat#60267-1-1), BAD (Cat#10435-1-AP), Bcl-2 (Cat#12789-1-AP), Bcl-xl (Cat#10783-1-AP), Caspase3/P17/P19 (Cat#19677-1-AP), Caspase8/P43/P18 (Cat#13423-1-AP), and P21 (Cat#10355-1-AP) were purchased from Wuhan, China. Antibody actin (Cat#21338-1) was purchased from SAB, China. Acetyl-Histone H3 (Cat#AF1684), Acetyl-Histone H4 (Cat#AJ4218), Phos-AKT1 (Cat#AF1546), CDK4 (Cat#AF2515), Cyclin D1 (Cat#AF0126), and Phos-RB1 (Cat#AF1135) were purchased from Beyotime International, Nantong, China. H3 (Cat#A2348) and Caspase3 (A19653) were purchased from ABclonal, Wuhan, China. Cleaved-Caspase9 (Cat#52873T) was purchased from Cell Signal Technology, Danvers, MA, USA.

### 2.10. Data Processing and Statistical Analyses

GraphPad Prism 9.0.0 for Windows (San Diego, CA, USA) was applied for statistical analysis. One-way ANOVA was performed. *p*-Values < 0.05 were considered statistically significant. Apoptosis and cycle results were analyzed using Flowjo V10 software (BD Life Sciences). Protein expression levels were quantified using (Fiji Is Just) ImageJ 2.9.0 software (https://fiji.sc/) (accessed 26 August 2023). The protein search library used Proteome Discoverer 2.3. GO analysis of proteomics data was performed using the DAVID (https://davidbioinformatics.nih.gov/) (accessed 26 August 2023), and the results were then visualized using https://www.bioinformatics.com.cn/ (accessed 26 August 2023). Protein–Protein Interaction Networks were obtained from the STRING database (https://cn.string-db.org/) (accessed 31 August 2023) and visualized by Cytoscape 3.7.2.

### 2.11. Molecular Docking

Molecular docking of NBU-1, NBU-2, and SAHA with HDAC1 and HDAC6 was performed using the CDOCKER protocol in Discovery Studio 2020. Crystal structures of HDAC1 (PDB: 5ICN) and HDAC6 (PDB: 6UO2) were obtained from the RCSB Protein Data Bank. Protein preparation involved removing water molecules, adding hydrogens, and setting protonation states at pH 7.4 via the “Prepare Protein” tool. Ligand structures were built and prepared using the “Prepare Ligands” module at pH 7.4. The conformation with the lowest CDOCKER Interaction Energy was selected as the most favorable binding pose.

## 3. Results

### 3.1. Chemical Synthesis

**Scheme 1:**
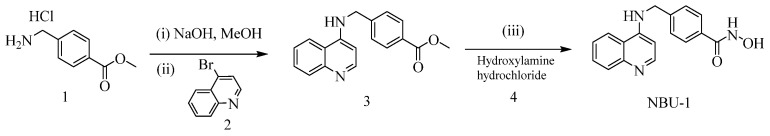
The synthetic route for NBU-1. Reaction reagents and conditions: (i) NaOH, anhydrous methanol, ice water bath, 1 h; (ii) Palladium acetate, DPEPhos, potassium phosphate, 1,4-dioxane, 100 °C, 10–12 h; (iii) Sodium tert-butoxide, hydroxylamine hydrochloride, methanol, 0 °C, 20 min, then room temperature for 2–3 h.

**Scheme 2:**
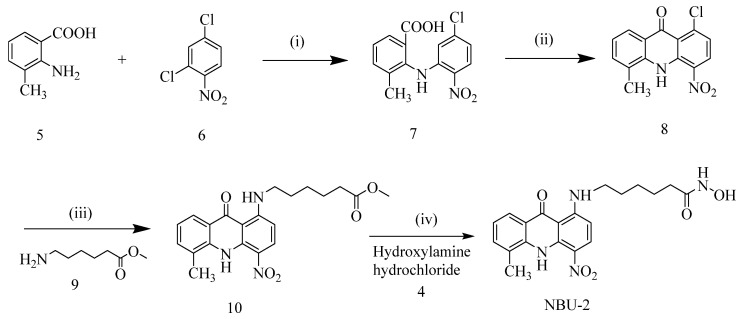
The synthetic route for NBU-2. Reaction reagents and conditions: (i) DMF, K_2_CO_3_, Cu powder, 130 °C, overnight; (ii) H_2_SO_4_, 80 °C, 4 h; (iii) 2-ethoxyethanol, methyl 6-aminohexanoate hydrochloride, Et_3_N, 90–100 °C, overnight; (iv) Hydroxylamine hydrochloride, sodium methylate, methanol, 0 °C, 30 min, then room temperature for 2–4 h.

The synthesis routes of NBU-1 and NBU-2 were described in Scheme 1 and Scheme 2. For the synthesis of NBU-1, methyl 4-(aminomethyl)benzoate hydrochloride **1** and 4-bromoquinoline **2** were used as starting materials, and intermediate **3** was obtained by *N*-nucleophilic substitution reaction. Finally, the target compound NBU-1 was obtained by reacting with hydroxylamine hydrochloride **4**; For the synthesis of NBU-2, using 2-amino-3-methylbenzoic acid **5** and 2,4-dichloro-1-nitrobenzene **6** as starting materials, the benzoic acid **7** was obtained through Cu-catalyzed Ullmann reaction. Friedel-Crafts acylation in concentrated sulfuric acid afforded acridone **8**, which was transformed into intermediate **10** via *N*-nucleophilic substitution with amine **9**. Subsequent reaction with hydroxylamine hydrochloride **4** yielded NBU-2. The structures of all target compounds were verified by ^1^H NMR, ^13^C NMR, and high-resolution mass spectrometry (HRMS).

### 3.2. In Vitro HDAC Isoforms (HDAC1/6) Selectivity

For the enzymatic inhibition assays, SAHA was employed as a positive control and reference inhibitor. SAHA is an FDA-approved, pan-HDAC inhibitor that provides a universally recognized benchmark for potency. The inclusion of SAHA in all assays allows for a critical assessment of the relative inhibitory efficacy of our newly synthesized compounds against both HDAC1 and HDAC6 isoforms within the well-established context of a clinical agent. NBU-1, NBU-2, and the positive control SAHA were tested for their enzyme inhibitory activities against class I HDAC (HDAC1) and class II HDAC (HDAC6), to explore their potency and isoform selectivity (Table 1). The results displayed that NBU-2 had notable inhibitory activities against HDAC1 and HDAC6, with IC_50_ values of 7.75 nM and 7.34 nM, respectively, which were better than the positive control SAHA.

### 3.3. Binding Modes of NBU-1 and NBU-2 with Protein HDAC1/6

To further explain the difference in the inhibitory activity of NBU-1 and NBU-2 against HDACs, molecular docking was used to analyze the docking modes of small molecular ligands against different isoforms of HDAC. It can be seen from the results (Table 2) that NBU-2 has a lower CDocker interaction energy value and Binding energy value with both HDAC1 and HDAC6 compared to NBU-1 and SAHA. The well-characterized pan-HDAC inhibitor SAHA was included as a reference compound in our docking simulations. This approach served two key purposes: first, to validate our docking protocol by reproducing its established binding mode within the HDAC active site; and second, to enable a direct, head-to-head comparison of the predicted binding interactions and affinities of our novel conjugates (NBU-1 and NBU-2) against a clinical benchmark.

**Figure 1:**
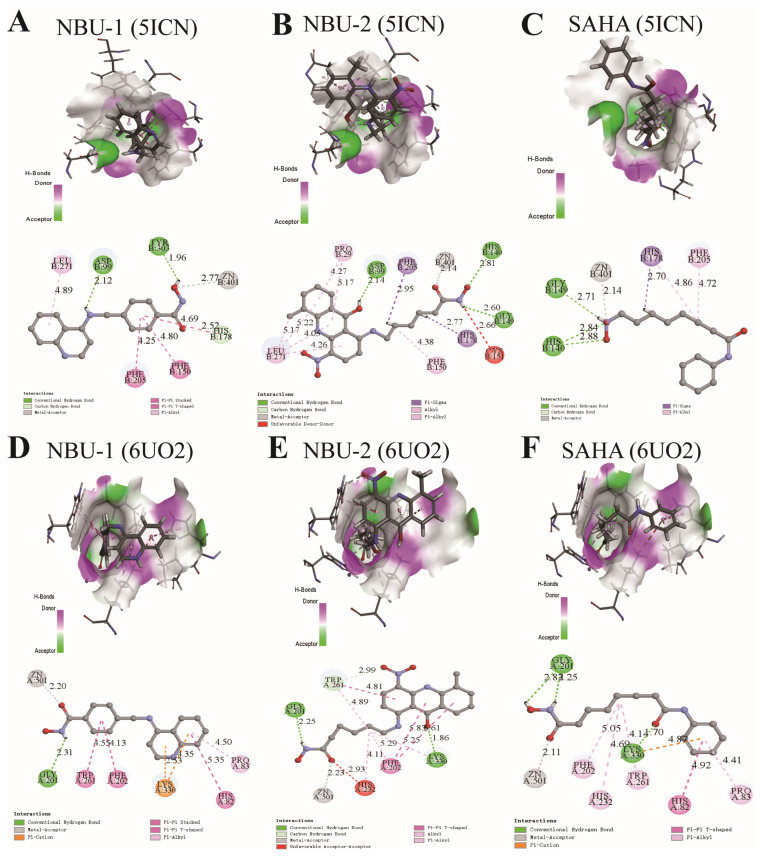
The binding modes of NBU-1, NBU-2, and SAHA with HDAC1 (PDB ID: 5ICN) and HDAC6 (PDB ID: 6UO2). (**A**) The binding modes of NUB-1with HDAC1. (**B**) The binding modes of NUB-2 with HDAC1. (**C**) The binding modes of SAHA with HDAC1. (**D**) The binding modes of NUB-1with HDAC6. (**E**) The binding modes of NUB-2 with HDAC6. (**F**) The binding modes of SAHA with HDAC6 The carbon atoms of compounds and amino acid residues are presented in cyan and gray, respectively. Oxygen and nitrogen atoms are colored red and blue, respectively. The zinc ion is shown as a gray sphere. Non-bonded interactions are indicated by dashed lines, with colors designating different interaction types.

Molecular docking analysis of NBU-1 with HDAC1 (PDB ID: 5ICN, Figure 1A) revealed that the hydroxyl group of the hydroxamic acid moiety coordinated with the Zn^2+^ ion, while also forming a hydrogen bond with Tyr303. The amino group at the 4-position of the quinoline ring formed another hydrogen bond with Asp99. In addition, the benzene ring and quinoline ring formed multiple Pi-Pi interactions with Phe205, Phe150, His178, and a Pi-alkyl interaction with Leu271. Compared with NBU-1, NBU-2 had more interactions with HDAC1 protein (Figure 1B). In addition to forming a coordination bond with the zinc ion, the hydroxamic acid group also interacted with His140 and Gly149 through two hydrogen bonds. The planar conjugated aromatic acridone scaffold in NBU-2 was situated within a hydrophobic region, enabling the formation of several Pi-alkyl interactions with Leu271 and Pro29. In addition, the aliphatic flexible linker also formed multiple Pi-sigma and Pi-alkyl interactions with residues Phe205, Phe150, and His178. The binding modes of NBU-1 and NBU-2 against HDAC6 protein (PDB ID: 6UO2) were similar, as shown in Figure 1D,E. It should be noted that NBU-2 formed two hydrogen bonds with Gly201 and Lys330, and a hydrocarbon bond with Trp261, which showed more advantages than NBU-1. As a control, the docking results of SAHA with HDAC1 and HDAC6 were shown in Figure 1C,F, respectively.

### 3.4. Anti-Proliferative Properties of NBU-2 Against Various Types of Tumor Cells

Tumor cells are characterized by their ability to proliferate without restriction, so inhibiting their growth is a key direction in the development of anti-cancer drugs. To identify the most sensitive cancer type to NBU-2 and to provide a rationale for subsequent in-depth mechanistic studies, we investigated the effectiveness of NBU-2 in inhibiting the proliferation of various tumor cells (Jurkat, K562, HL-60, CCRF-CEM, U937, HCT-116, A549, Hep G2, MDA-MB-231, MCF-7, 4T1, PC12) within 72 h. The MTT and CCK-8 assays were used for this purpose. We also examined the toxicity of NBU-2 on normal cells (HUVEC). The IC_50_ of each cell was summarized in Table 3.

From the experimental data in Table 3, it can be seen that NBU-2 has an inhibitory effect on a wide range of tumor cells. Strikingly, it demonstrated superior potency against Jurkat cells (a model for T-cell acute lymphoblastic leukemia, T-ALL), with an IC_50_ of 0.86 ± 0.13 μM at 72 h, which was superior to the IC_50_ of 72 h of Chidamide (2.23 μM) and only slightly lower than that of SAHA (0.37 μM). Notably, the inhibitory effect of NBU-2 on other hematological malignancies such as HL-60, CCRF-CEM, and U937 was also more obvious, with IC_50_ values below 5 μM. It also demonstrated some degree of inhibition on certain types of solid tumor cells. Based on these findings, we selected the most NBU-2-sensitive Jurkat (T-ALL) cell line for all subsequent mechanistic investigations to delve into the specific anti-leukemic mechanisms.

**Figure 2:**
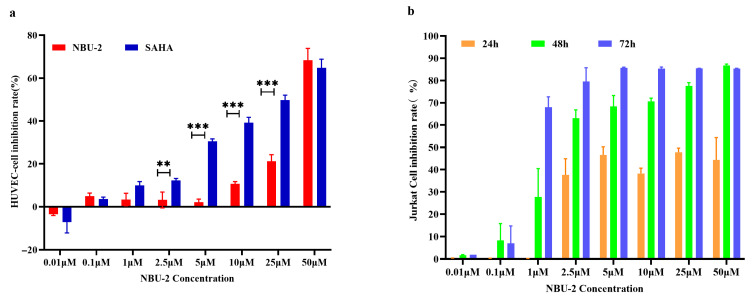
NBU-2 on the proliferation of HUVEC and Jurkat cells. (**a**) Cell viability through the use of an MTT assay. HUVECs were treated with different concentrations of NBU-2 (0.01, 0.1, 1, 2.5, 5, 10, 25, and 50 µM) for 72 h. (**b**) Cell viability through the use of a CCK-8 assay. Jurkat cells were treated with NBU-2 for different amounts of time. ** *p* < 0.01, *** *p* < 0.001.

We know that many medications can kill cancer cells, but also harm healthy cells with side effects. To verify its toxicity on HUVEC, we conducted a CCK8 assay on NBU-2 for 72 h. The results in Figure 2a demonstrate that NBU-2 exhibits less potent anti-proliferative activity against HUVECs (IC_50_ = 35.56 μM) than SAHA (IC_50_ = 5.62 μM). This marked difference in cytotoxicity toward normal cells highlights a potentially more favorable safety profile for NBU-2, which is a significant advantage for its further development as an anti-cancer agent. Furthermore, to characterize the anti-T-ALL activity in detail, when we compared the inhibitory effect of NBU-2 on different types of tumor cells, we found that it had the most pronounced inhibitory effect on Jurkat after 72 h. To investigate whether the inhibitory effect of NBU-2 on Jurkat cells varies with time change, we set up a time gradient of 24 h, 48 h, and 72 h to verify the time dependence of the inhibitory effect of NBU-2. Figure 2b presents the inhibition rate of NBU-2 on Jurkat cells at various concentrations and times. The inhibitory effect of NBU-2 on Jurkat cells was concentration and time-dependent, as shown by experimental results.

We noticed that the antiproliferative activity of NBU-2 appeared to be weaker compared to the enzyme activity. This can be caused by several factors, such as poor periplasmic properties of the compound, inability to enter the cell, binding of the compound to plasma proteins leading to a decrease in the free drug concentration, and the possibility that there may be other substances in the cell that bind to the target protein with high intensity and prevent drug binding, and so on. We presented the ADME (Absorption, Distribution, Metabolism, and Excretion) properties of NBU-2 in Supplementary Data S1 (ADME).

### 3.5. Whole Proteomic Analysis of Jurkat Cells Stimulated with NBU-2

**Figure 3:**
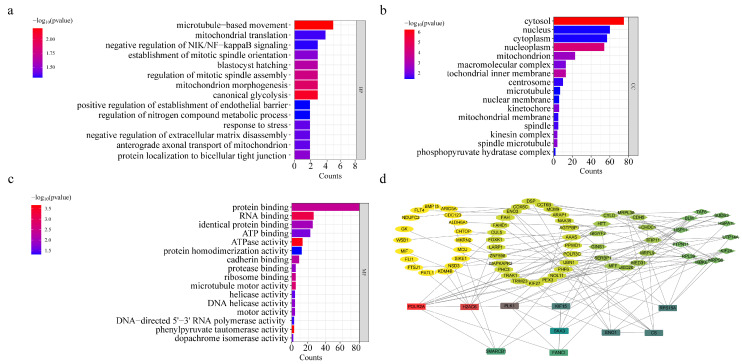
Graphs of GO analysis results and heatmap of differential genes. (**a**) GO-enriched barplot of BP. (**b**) GO-enriched barplot of CC. (**c**) GO-enriched barplot of MF. (**d**) Protein–Protein Interaction Networks, PPI.

To comprehensively explore the potential mechanism of NBU-2’s inhibition of Jurkat cell proliferation, we conducted a proteomic analysis of Jurkat cells stimulated with NBU-2 for 48 h. This time point was selected to capture the early mechanistic events of NBU-2 action while ensuring a sufficient number of viable cells for high-quality protein extraction, thereby avoiding the extensive secondary effects and cell lysis associated with longer incubation times (e.g., 72 h). Jurkat cells were stimulated using two concentrations of NBU-2, 1 μM and 5 μM, using the DMSO group as a control, and the experiment was repeated in triplicate for each group. Raw mass spectrometry data consisting of a large amount of peptide information was searched for libraries using Proteome Discoverer 2.3. 4085 proteins were obtained by searching the library. Excluding incidental values, 3292 proteins remain (detailed data displayed in Supplementary Data S2 (Proteomics)). We applied two screening conditions, *p* < 0.05 and |Log2 FC| > 1, resulting in 145 differentially expressed proteins that had the same trend in the 1 μM group and 5 μM group. Among these, 64 proteins were up-regulated after drug treatment, while 81 proteins were down-regulated. By importing these differentially expressed proteins into the DAVID database, we obtained the corresponding genes and the results of the GO enrichment analysis, which were then visualized through the https://www.bioinformatics.com.cn/ website (accessed 26 August 2023) to obtain the GO analysis map (Figure 3a–c).

It is well known that GO enrichment analysis consists of Biological Process (BP), Cell Component (CC), and Molecular Function (MF). We can see from the BP results graph that NBU-2 primarily affects the typical glycolytic processes, as well as the regulation of tumor necrosis factor-mediated signaling pathway and microtubule-based movement in cells. The results of CC suggested to us that NBU-2 acted mainly in the cytoplasm, nucleoplasm, and inner mitochondrial membrane. The MF results implied that NBU-2 was associated with ATPase activity, protein binding, and RNA binding.

The protein–protein interaction networks of these differential proteins were obtained using the STRING database. Using the cytoHubba plug-in in Cytoscape 3.7.2 software and the Degree algorithm was selected for the topology algorithm, the top 10 key protein genes were obtained as POLR2A, H2AC6, PLK1, CS, KIF15, RPS15A, ENO1, SKA3, FANCI, and SMARCB1, which are displayed in Figure 3d. These key protein genes mainly work in the cytoplasm, nucleoplasm, and mitochondria [19,20,21,22,23], which is in agreement with the results of GO analysis. Interestingly, these key proteins were strongly related to AKT, and both H2AC6 and FANCI were associated with histones [24,25,26]. These provided us with ideas for our next experiments—to explore the association of NBU-2 with histone modification and whether it affected intracellular AKT levels. In addition, they were closely related to apoptosis and the cell cycle [27,28]. These also further our thinking about the relationship between NBU-2 and apoptosis and the effect on the cell cycle.

### 3.6. The Presence of NBU-2 Affected the Level of Histone Acetylation

**Figure 4:**
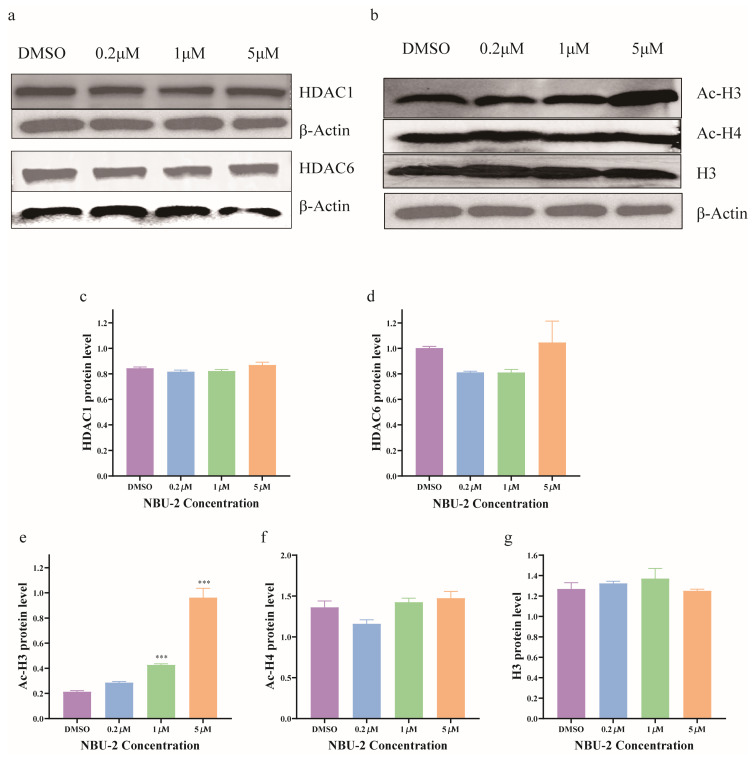
NBU-2 effects in Jurkat cells. (**a**) Western blotting analysis of HDAC1 and HDAC6. (**b**) Western blotting analysis of acetyl-H3, acetyl-H4, and H3 levels. (**c**) HDAC1 protein level. (**d**) HDAC6 protein level. (**e**) Ac-H3 protein level. (**f**) Ac-H4 protein level. (**g**) H3 protein level. Data are shown as the mean ± SD from three independent experiments (n = 3). *** *p* < 0.001.

It has been well established that HDAC is aberrantly expressed in many tumor cells; however, this abnormality is not consistent across tumors [29]. The results of proteomics showed that NBU-2 did not affect intracellular HDAC1 and HDAC6 protein expression. Western blot experiments were used to investigate this phenomenon. We could see that the HDAC1 and HDAC6 protein expression levels in Jurkat cells were not affected after NBU-2 treatment of Jurkat cells for 48 h (Figure 4).

SAHA has been shown to upregulate intracellular histone H3 acetylation levels by inhibiting HDAC enzyme activity, and numerous new studies have been published designing HDAC inhibitors that also exhibit the ability to increase the expression of Ac-H3 or Ac-H4 [30]. Among the whole proteomics results, we found that both H2AC6 and FANCI were correlated with histone modifications [24,26]. In conjunction with the existing reports, we discussed the role of NBU-2 in the regulation of chromatin core histones by detecting the levels of intracellular Ac-H3 and Ac-H4 protein expression. As shown in Figure 4, the intracellular Ac-H3 protein level was significantly increased after treatment with NBU-2 (48 h) in a concentration-dependent manner in Jurkat cells. However, there was no significant change in the Ac-H4 level, and the total histone H3 was not affected by the NBU-2 stimulation.

### 3.7. NBU-2 Inhibited Akt Phosphorylation in Jurkat Cells

**Figure 5:**
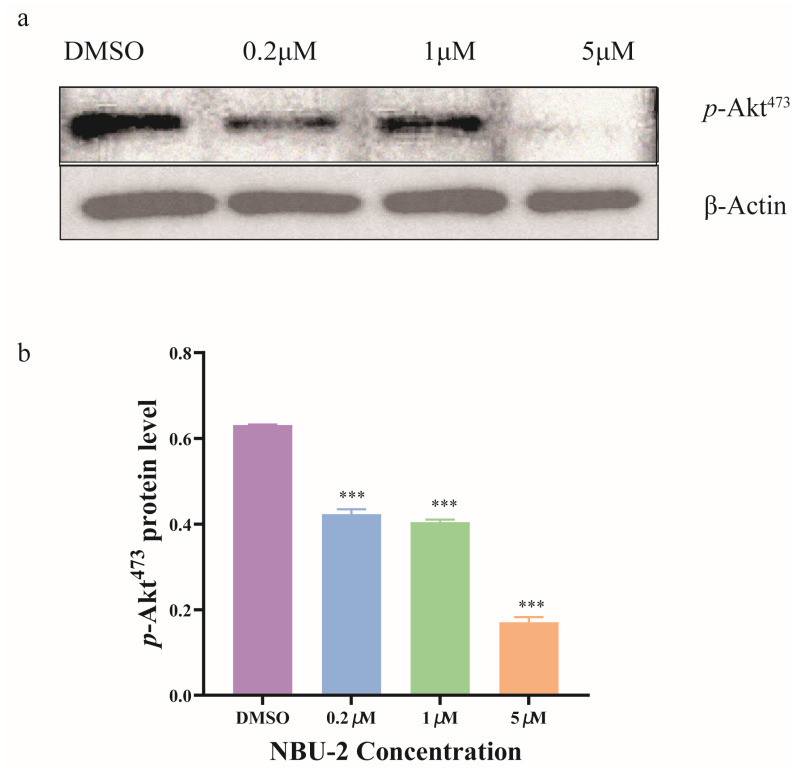
Effects of NBU-2 in Jurkat cells treated with NBU-2. (**a**) Western blotting analysis of *p*-Akt. (**b**) *p*-Akt Protein level. Data are shown as the mean ± SD from three independent experiments (n = 3). *** *p* < 0.001.

From the whole proteomics data, many of the key proteins were connected with AKT1 to different degrees. In turn, Akt is an extremely important part of the human signaling pathway [24,31]. Akt, also known as protein kinase B (PKB), which is one of the key proteins downstream of PI3K, is phosphorylated by PI3K and can enter into the nucleus and the cytoplasm, resulting in the phosphorylation of a series of substrates, which play a role in the cell’s regulation of cell death and proliferation and chemotherapy tolerance, thereby affecting the proliferation, survival, cycle, and growth of tumor cells [32]. It has been proposed that quisinostat, a pan-HDAC inhibitor, could down-regulate the level of *p*-Akt (Ser473) [33]. As shown in Figure 5 shown that NBU-2 inhibited Akt phosphorylation and significantly reduced the protein level of *p*-Akt (Ser473). As the concentration of NBU-2 increased, the protein expression of *p*-Akt (Ser473) was subsequently reduced, implying that the path of Akt phosphorylation pathway was blocked. As for the total Akt protein, we found that among the numerous proteins obtained by whole proteomics, it was detected in both the control (DMSO group) and experimental (1 µM and 5 µM groups), and it did not show any significant change (*p* > 0.05).

### 3.8. NBU-2 Induced Apoptosis in Jurkat Cells

**Figure 6:**
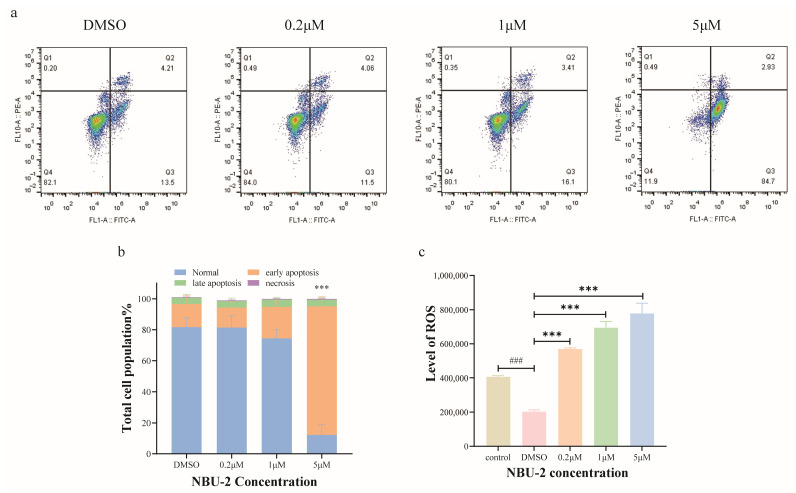
NBU-2 induces apoptosis in Jurkat cells. (**a**) Apoptosis in Jurkat cells; (**b**) Percentage of apoptotic cells at each stage of apoptosis. (**c**) Reactive oxygen detection. Data are shown as the mean ± SD from three independent experiments (n = 3). ^###^ *p* < 0.001, *** *p* < 0.001.

Apoptosis, a form of programmed cell death, can be activated by extrinsic or intrinsic pathways [34]. Annexin V-FITC and PI double staining is a highly effective method for identifying cells in various stages of apoptosis. We used an Annexin V-FITC Apoptosis Detection Kit to detect apoptosis. The results of the apoptosis assay after 48 h of treatment with NBU-2 are illustrated in Figure 6a. Based on the findings, when subjected to DMSO and 0.2 µM concentrations, the percentage of cells in the early apoptotic stage was 13.5% and 11.5%, respectively. At a concentration of 1 µM at NBU-2, the rate of early apoptosis was 16.1%. However, at a concentration of 5 µM, the number of cells in a prematurely apoptotic state had surged to 84.7%. These experimental data revealed that NBU-2 induced early apoptosis in Jurkat cells, and the induction showed a concentration-dependent effect.

When cells are damaged, they produce high levels of ROS, which in turn harm the mitochondrial membrane, ultimately resulting in apoptosis [35]. Hence, we performed a reactive oxygen species assay using the fluorescent probe DCFH-DA on Jurkat cells treated with NBU-2 for 48 h. The experimental results were displayed in Figure 6c, with DMSO as a blank control for reactive oxygen species. We found that after Jurkat cells were treated with NBU-2 for 48 h, the levels of reactive oxygen species in all three groups, 0.2 µM, 1 µM, and 5 µM, were significantly increased relative to the DMSO blank control group in a concentration-dependent manner. This phenomenon suggested that NBU-2 could induce apoptosis by inducing cells to produce large amounts of reactive oxygen species.

In the results of proteomics GO enrichment analyses, NBU-2 was shown to be closely associated with mitochondria and could significantly affect ATPase activity. It has been shown that mitochondrial fission causes reactive oxygen species accumulation and affects ATPase activity [36], which corroborates with the experimental results of NBU-2-induced reactive oxygen species accumulation, making our experimental results more credible.

### 3.9. NBU-2 Induced Endogenous Apoptosis in Jurkat Cells

**Figure 7:**
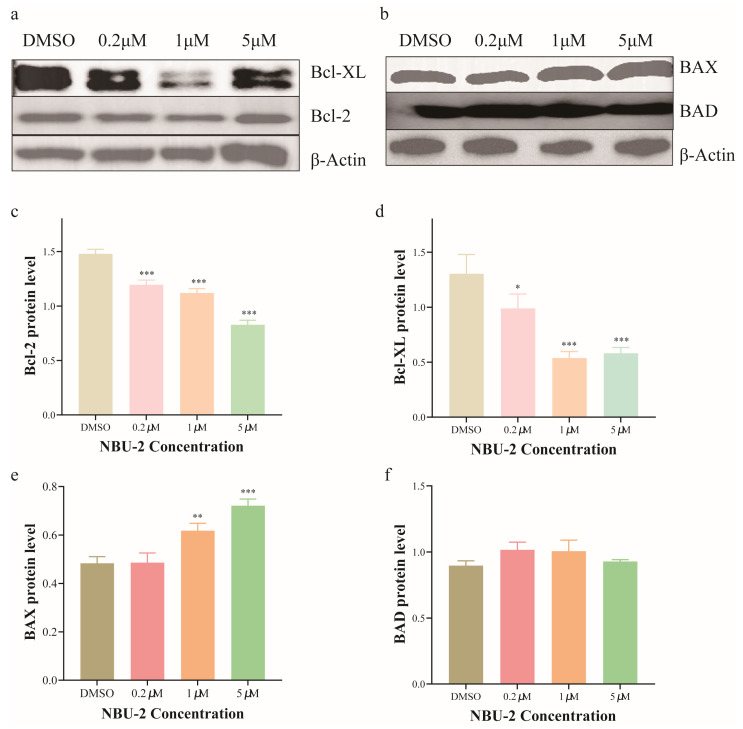
Expression of Bcl-2 family proteins. (**a**) Western blotting analysis of Bcl-2 and Bcl-XL. (**b**) Western blotting analysis of BAX and BAD. (**c**) Bcl-2 protein level. (**d**) Bcl-XL protein level. (**e**) BAX protein level. (**f**) BAD protein level. Data are shown as the mean ± SD from three independent experiments (n = 3). * *p* < 0.05, ** *p* < 0.01, *** *p* < 0.001.

Bcl-2 family proteins are essential in apoptosis, where they are found in the endoplasmic reticulum and mitochondrial outer membrane [37,38]. Bcl-2 and Bcl-XL are two prominent members of this family, which act as “protectors” and possess anti-apoptotic functions. Whereas BAX is mainly located in the cytoplasm and acts as an effector to promote apoptosis (Pro-apoptotic) [39]. BAD, on the other hand, indirectly initiates apoptotic signaling by binding to the anti-apoptotic Bcl-2 protein, thus preventing it from controlling the pro-apoptotic BAX and BAK [40,41]. Figure 7 shows the expression of Bcl-2 family proteins in Jurkat cells after NBU-2 treatment, respectively. For the anti-apoptotic proteins Bcl-2 and Bcl-XL, the experimental results showed the same phenomenon, in whcih the expression of these two proteins was reduced by the effect of NBU-2. In turn, the pro-apoptotic protein BAX increased with the concentration of NBU-2, in contrast to BAD, which was unaffected by NBU-2. From this, we concluded that NBU-2 is inducing Jurkat cells to perform an apoptotic program by stimulating an increase in the expression of the pro-apoptotic protein BAX and a decrease in the expression of the pro-survival protein Bcl-2 and Bcl-XL.

**Figure 8:**
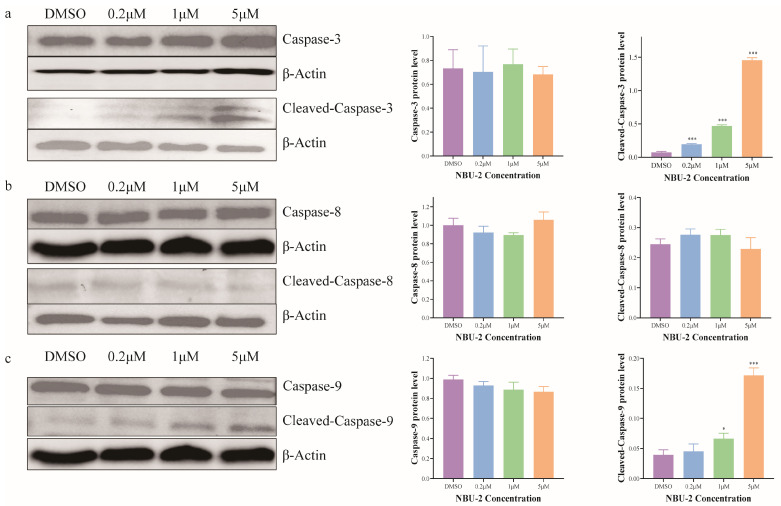
Expression of Caspase family proteins. (**a**) Western blotting analysis of Caspase-3 and Cleaved-Caspase-3. (**b**) Western blotting analysis of Caspase-8 and Cleaved-Caspase-8. (**c**) Western blotting analysis of Caspase-9 and Cleaved-Caspase-9. Data are shown as the mean ± SD from three independent experiments (n = 3). * *p* < 0.05, *** *p* < 0.001.

The exogenous pathway (death receptor pathway) and the endogenous pathway (also known as the mitochondrial or BCL-2 regulatory pathway) are two triggering mechanisms for apoptosis. The exogenous pathway is induced by the activation of the death receptor on the plasma membrane, which induces the activation of caspase-8. Caspase-9 activation, on the other hand, is an endogenous pathway induced intracellularly by various stress stimuli. Once the initiators Caspase-8 and Caspase-9 are activated, endogenous and exogenous apoptosis share common pathway components, Cleaved-Caspase-3 and Cleaved-Caspase-7, which act as executioners to perform various apoptotic actions [42]. Hence, we also examined the expression of Caspase-8, 9, 3 and Cleaved-Caspase-8, 9, 3 proteins using Western blot. The results of the experiments were demonstrated in Figure 8, which showed that NBU-2 did not affect the expression of the three prototypes of Caspase-8, 9, and 3, whereas the expression of Cleaved-Caspase-9 and Cleaved-Caspase-3 increased with the increase in the concentration of NBU-2. This phenomenon indicated that NBU-2 induced apoptosis through an endogenous pathway.

### 3.10. Effect of NBU-2 on the Induction of Cell-Cycle Arrest

**Figure 9:**
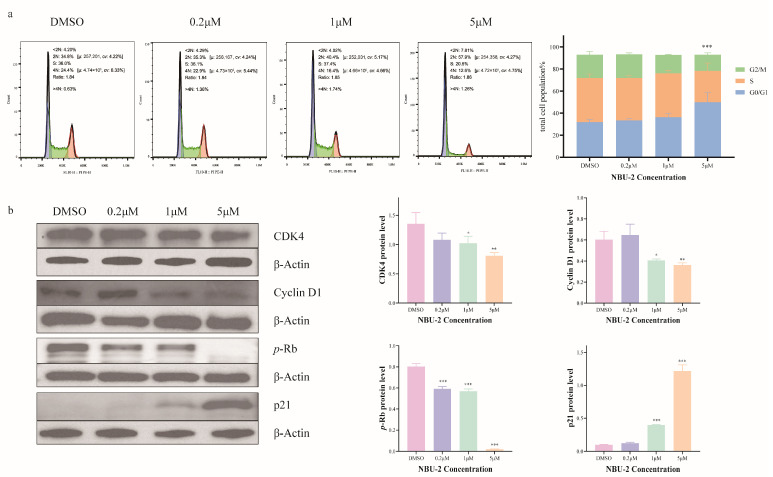
Effect of NBU-2 on the induction of cell-cycle arrest. (**a**) Flow cytometry analysis of the cell cycle. (**b**) Western blotting analysis of Cell cycle-related proteins. (CDK4, Cyclin D1, *p*-Rb and p21). Data are shown as the mean ± SD from three independent experiments (n = 3). * *p* < 0.05, ** *p* < 0.01, *** *p* < 0.001.

The cell cycle is a series of events that must occur during cell division and then proliferation. We determined the effect of NBU-2 on Jurkat cells by flow cytometry combined with propidium iodide staining solution from the Cell Cycle and Apoptosis Kit. The results showed that after stimulating Jurkat cells with DMSO and a low concentration of 0.2 μM of NBU-2 for 48 h, the percentage of G0/G1 phase was 34.8% and 35.3%, respectively. When the concentration of NBU-2 was increased to 1 μM, the percentage of cells in the G0/G1 phase also increased to 40.4%, and by increasing the concentration of NBU-2 to 5 μM, the percentage of cells in the G0/G1 phase increased to 57.9% (Figure 9a). The results of cell cycle experiments in Figure 9a implied that NBU-2 induced G0/G1 phase block in Jurkat cells in a concentration-dependent manner.

The normal progression of the cell division cycle depends on the level of expression of the protein family of cell cycle proteins (cyclins) and the subsequent activation of cyclin-dependent kinases (CDKs) [43]. Cyclin D1, the G1/S-specific cyclin-D1, normally binds to CDK4/6 to form a complex that is indispensable for the transition of the cell cycle from the G1 to the S phase [44]. It has been shown that CDK4 interacts with Cyclins to form a complex and then phosphorylates the downstream Rb (Retinoblastoma) protein to promote the transition from the G0/G1 phase to the S phase [45]. p21 is the major CDK inhibitor, which directly inhibits DNA replication and prevents the transition from the G0/G1 to the S phase [46]. Histone deacetylases (HDACs) have inhibitory effects on p21, while HDAC inhibitors upregulate p21 expression [47]. In order to investigate the molecular mechanism by which NBU-2 blocks the progression of Jurkat cells from the G0/G1 phase to the S phase, we examined the expression of these proteins using a Western blot. As demonstrated in Figure 9b, Cyclin D1 and CDK4 expression were inhibited in Jurkat cells treated with NBU-2 for 48 h in a concentration-dependent manner. Equally, as the concentration of NBU-2 increased, the expression of *p*-Rb decreased. Proteomic results showed that the expression of Rb was not affected by NBU-2. As an HDAC inhibitor, NBU-2 increased the expression of p21 in a concentration-dependent manner. These results suggested that the inhibition of HDAC activity by NBU-2 relieved the inhibitory effect of HDAC on p21, thereby inhibiting the expression of CDK 4 and Cyclin D1, and subsequently inhibiting the phosphorylation of the downstream Rb protein, and finally arresting the cells in G0/G1 phase.

## 4. Discussion

Acute T-lymphoblastic leukemia (T-ALL) is classified as a high-risk acute lymphoblastic neoplasm due to its poor response to conventional chemotherapy and high relapse rate [1]. As HDAC inhibitors represent one of its important treatment approaches [3,7,8], the development of novel HDAC inhibitors is particularly necessary. This study identified the synthetic compound NBU-2 as a highly potent HDAC inhibitor, which outperformed the positive control SAHA by exhibiting IC_50_ values of 7.75 nM against HDAC1 (class I) and 7.34 nM against HDAC6 (class II). The molecular docking studies provided a structural rationale for this enhanced potency.

To better understand how HDAC inhibitors fight against tumors at the cellular level, we first investigated the effectiveness of NBU-2 in inhibiting the proliferation of a broad panel of tumor cell lines, with a particular emphasis on hematological malignancies such as Jurkat, HL-60, CCRF-CEM, and U937. It is important to acknowledge that while the Jurkat cell line serves as a well-established and widely utilized model for T-ALL research, providing valuable insights into disease mechanisms and drug response, it cannot fully recapitulate the genetic and functional heterogeneity of primary patient samples. Therefore, the promising effects of NBU-2 observed in Jurkat cells necessitate further validation in primary ALL blasts and a broader spectrum of leukemic cell lines in future investigations. Notably, NBU-2 exhibited exceptional activity against human acute T-cell leukemia Jurkat cells, with an IC_50_ value of 0.86 μM superior to Chidamide and approaching that of SAHA. The anti-proliferative effect on Jurkat cells was confirmed to be both concentration- and time-dependent. A critical finding was its significantly reduced toxicity towards normal HUVECs compared to SAHA, suggesting a potentially wider therapeutic window. We note that the use of HUVECs, while standard for initial toxicity screening, underscores the need to include a broader panel of primary cells or tissue-specific normal cells in future in vivo and translational studies to comprehensively assess safety.

To comprehensively investigate the underlying mechanism by which NBU-2 inhibited Jurkat cell proliferation, we conducted a proteomic analysis of Jurkat cells stimulated with NBU-2 for 48 h. This analysis identified 145 differentially expressed proteins, and GO enrichment suggested involvement in key processes like glycolysis, tumor necrosis factor signaling, and microtubule-based movement, primarily within the cytoplasm, nucleoplasm, and mitochondria. Furthermore, the identification of the top 10 key protein genes (POLR2A, H2AC6, PLK1, CS, KIF15, RPS15A, ENO1, SKA3, FANCI, and SMARCB1) and their association with AKT signaling, apoptosis, and the cell cycle provided crucial directions for subsequent mechanistic validation.

Further experiments elucidated the multifaceted mechanism by which NBU-2 inhibited proliferation and induced apoptosis in Jurkat cells. Contrary to affecting HDAC protein levels, NBU-2 functionally inhibited their activity, leading to a concentration-dependent increase in acetylated histone H3 (Ac-H3), a classic epigenetic marker of HDAC inhibition [30]. Mechanistically, NBU-2 was found to potently inhibit the phosphorylation of AKT at Ser473, thereby blocking this critical pro-survival signaling pathway. This triggered the intrinsic apoptotic pathway, as evidenced by a concentration-dependent surge in reactive oxygen species (ROS), a dramatic increase in early apoptosis (84.7% at 5 µM), and corresponding shifts in Bcl-2 family protein expression (decreased Bcl-2/Bcl-XL, increased BAX). The activation of the mitochondrial apoptosis pathway was confirmed by the cleavage of caspase-9 and caspase-3, but not caspase-8. Concurrently, NBU-2 induced a G0/G1 cell cycle arrest. This arrest was mechanistically linked to the upregulation of p21 (a known consequence of HDAC inhibition [47]) and the subsequent downregulation of Cyclin D1 and CDK4, leading to suppressed phosphorylation of Rb and a blockade in the G0/G1 to S phase transition. Our study focused on the upstream Cyclin D1/CDK4/Rb axis; however, future work will include analysis of the downstream E2F1/Cyclin E circuit to fully delineate the mechanism of cell cycle arrest. Collectively, NBU-2 exerts its potent anti-tumor effects through a coordinated mechanism involving epigenetic modification, inhibition of pro-survival AKT signaling, induction of ROS-mediated intrinsic apoptosis, and cell cycle arrest.

Finally, the translational potential of NBU-2 warrants discussion regarding its physicochemical properties. As a lipophilic compound, its in vivo delivery may present challenges such as poor aqueous solubility and rapid metabolism. While preliminary in silico ADME predictions are provided (Supplementary Datas), future studies must empirically address these pharmacokinetic aspects. Strategies such as formulation with liposomes, nanoparticles, or cyclodextrins could be explored to enhance its solubility, stability, and bioavailability, which will be crucial for evaluating its efficacy in animal models and beyond.

## 5. Conclusions

In summary, we synthesized two novel compounds designated NBU-1 and NBU-2, with NBU-2 demonstrating significant inhibitory effects against both HDAC1 and HDAC6 isoforms. The concurrent targeting of these distinct HDAC subtypes—nuclear HDAC1, primarily regulating histone acetylation and gene expression, and cytoplasmic HDAC6, involved in cell motility and protein degradation—likely contributes to the compound’s potent efficacy. Subsequent mechanistic investigations focused on NBU-2 revealed its broad-spectrum antiproliferative activity across multiple tumor cell lines. This compound inhibits HDAC1 and HDAC6, leading to increased histone H3 acetylation levels and suppression of AKT phosphorylation, ultimately activating the intrinsic apoptosis pathway. Cell cycle analysis further identified that NBU-2 effectively blocked cell progression from the G0/G1 phase to the S phase. Whole proteomic profiling corroborated these mechanistic observations. This work established a foundation for developing innovative anti-cancer therapeutics and investigating their mechanism-specific pharmacological actions.

## Figures and Tables

**Table 1:** Inhibitory activities of NBU-1 and NBU-2 against histone deacetylases.

Compound	IC_50_ ^a^ (nM)	
	HDAC1	HDAC6
NBU-1	242 ± 15	114 ± 28
NBU-2	7.75 ± 0.57	7.34 ± 0.60
SAHA	17 ± 1.19	15 ± 0.89

Note: ^a^ IC_50_: mean ± SEM of three independent assays.

**Table 2:** Molecular docking of NBU-1, NBU-2, and SAHA against HDAC1/6.

Protein (PDB ID)	Compound	CDocker Interaction Energy ^a^ (kcal/mol)	Binding Energy ^b^ (kcal/mol)
HDAC1 (5ICN)	NBU-1	33.4941	−94.1843
	NBU-2	53.7173	−151.612
	SAHA	41.8092	−118.089
HDAC6 (6UO2)	NBU-1	51.2669	−121.675
	NBU-2	57.6625	−143.419
	SAHA	53.1881	−142.896

Note: ^a^ A scoring function in Discovery Studio 2020 used to predict ligand-target binding affinity. Higher values indicate stronger binding. ^b^ Represents the binding free energy between the ligand and protein receptor; lower values correspond to more favorable binding.

**Table 3:** IC_50_ values for NBU-2 inhibition of the proliferation of various tumor cells and normal cells.

Cell Lines	IC_50_ ^a^ (nM)		
	NBU-2	SAHA	Chidamide
Jurkat	0.86 ± 0.13	0.37 ± 0.12	2.23 ± 0.22
K562	7.02 ± 1.85	18.20 ± 4.50	1.65 ± 0.64
HL-60	1.92 ± 0.08	3.04 [15]	0.86 ± 0.07
CCRF-CEM	2.68 ± 0.30	0.37 ± 0.20	1.66 ± 0.34
U937	2.34 ± 1.08	0.97 ± 0.12	1.31 ± 0.38
HCT-116	7.06 ± 1.48	2.50 [16]	3.86 ± 1.09
A549	33.70 ± 2.15	1.80 [17]	>50
HepG2	7.55 ± 0.46	3.33 [18]	0.50 ± 0.02
MDA-MB-231	8.76 ± 1.88	1.34 ± 0.16	9.75 ± 1.78
MCF-7	14.91 ± 3.21	0.97 ± 0.35	- ^b^
4T1	>50	>50	>50
PC12	>50	>50	>50
HUVEC	35.56 ± 7.29	5.62 ± 1.46	- ^b^

Note: ^a^ IC_50_: mean ± SEM of three independent assays; ^b^ Not tested.

## Data Availability

Data are contained within the article and Supplementary Materials.

## Supplementary Materials

The following supporting information can be downloaded at: https://www.mdpi.com/article/10.3390/cells14221822/s1.
