# Supplementary material for: Novel Nitrogen Heterocycle–Hydroxamic Acid Conjugates Demonstrating Potent Anti-Acute Lymphoblastic Leukemia Activity: Induction of Endogenous Apoptosis and G0/G1 Arrest via Regulation of Histone H3 Acetylation and AKT Phosphorylation in Jurkat Cells
Source: Cells. 2025 Nov 20;14(22):1822. doi: 10.3390/cells14221822 (PMC12651750; doi:10.3390/cells14221822)
Supplement: Supplementary file 1 [file cells-14-01822-s001.zip › cells-3960132-supplementary/Supplementary Materials/Highlights.docx]

## Highlights

- **NBU-2** inhibited HDAC1/6 with IC_50_ values of 7.75 nM and 7.34 nM, respectively.
- The cellular mechanism of **NBU-2** is associated with AKT and histones.
- **NBU-2** promoted apoptosis in Jurkat cells through the endogenous pathway.
- **NBU-2** blocked cell progression from the G0/G1 to the S phase.
