# Supplementary material for: Novel Nitrogen Heterocycle–Hydroxamic Acid Conjugates Demonstrating Potent Anti-Acute Lymphoblastic Leukemia Activity: Induction of Endogenous Apoptosis and G0/G1 Arrest via Regulation of Histone H3 Acetylation and AKT Phosphorylation in Jurkat Cells
Source: Cells. 2025 Nov 20;14(22):1822. doi: 10.3390/cells14221822 (PMC12651750; doi:10.3390/cells14221822)

Fig. S4 NBU-2 effects in Jurkat cells. (**a**) Western blotting analysis of HDAC1 and HDAC6.

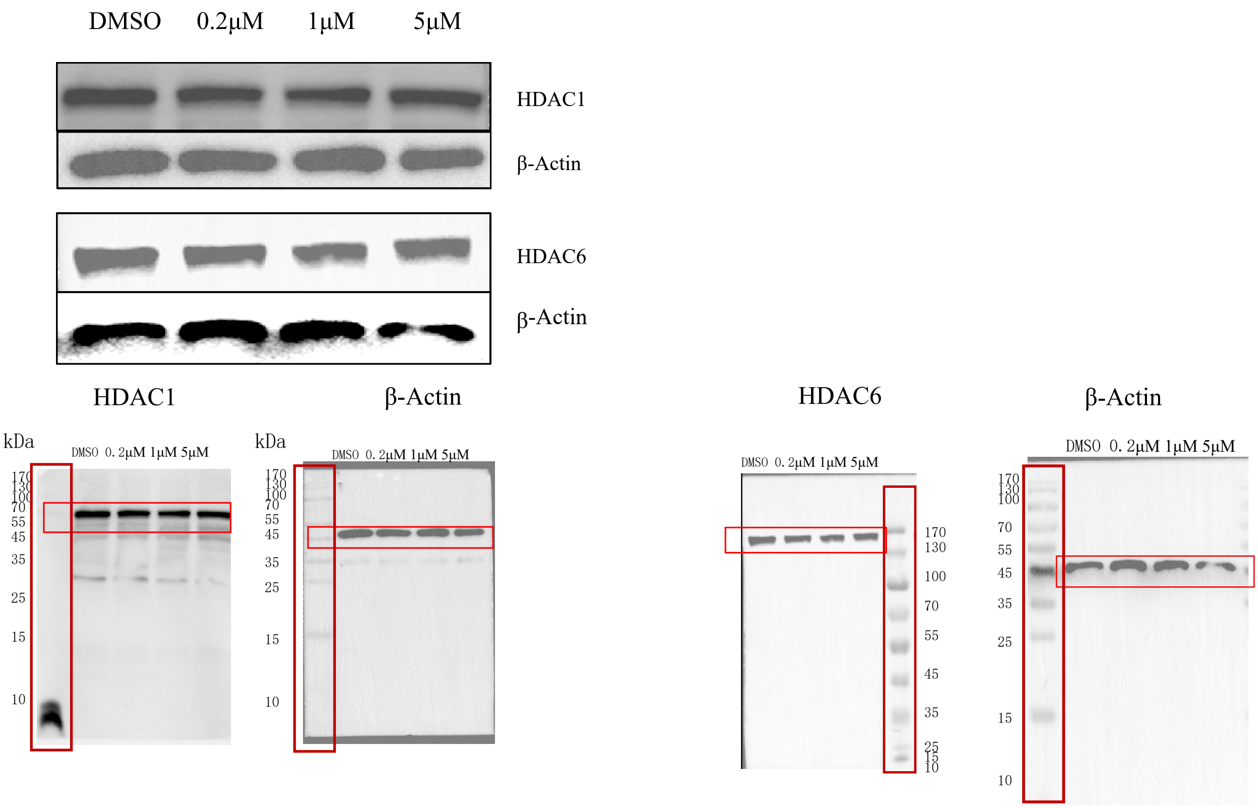

Fig. S4 NBU-2 effects in Jurkat cells. (**b**) Western blotting analysis of acetyl-H3, acetyl-H4 and H3 levels.

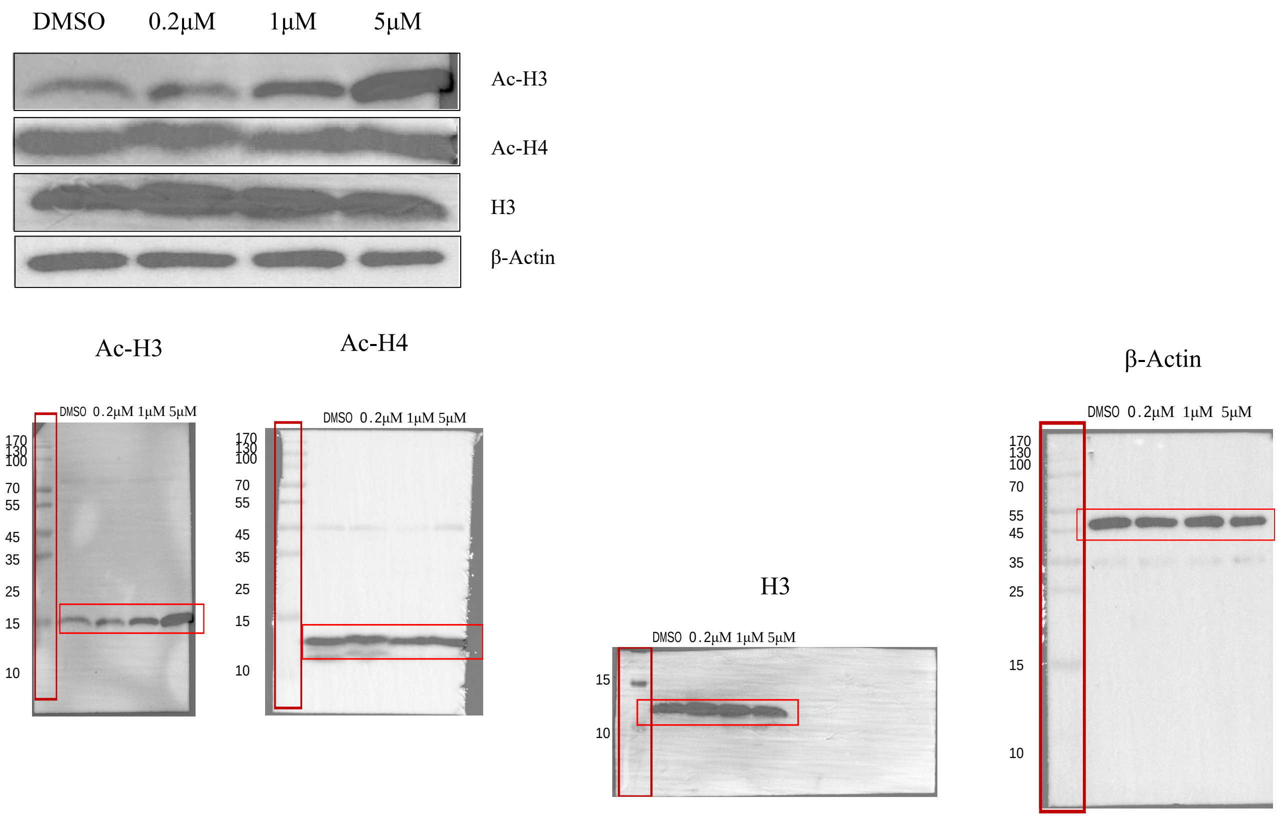

Fig. S5 Effects of NBU-2 in Jurkat cells treated with NBU-2. (a) Western blotting analysis of 431 p-Akt.

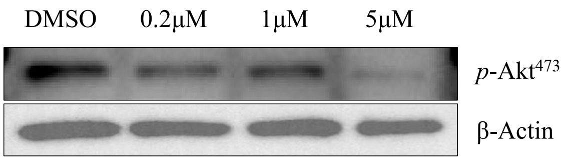

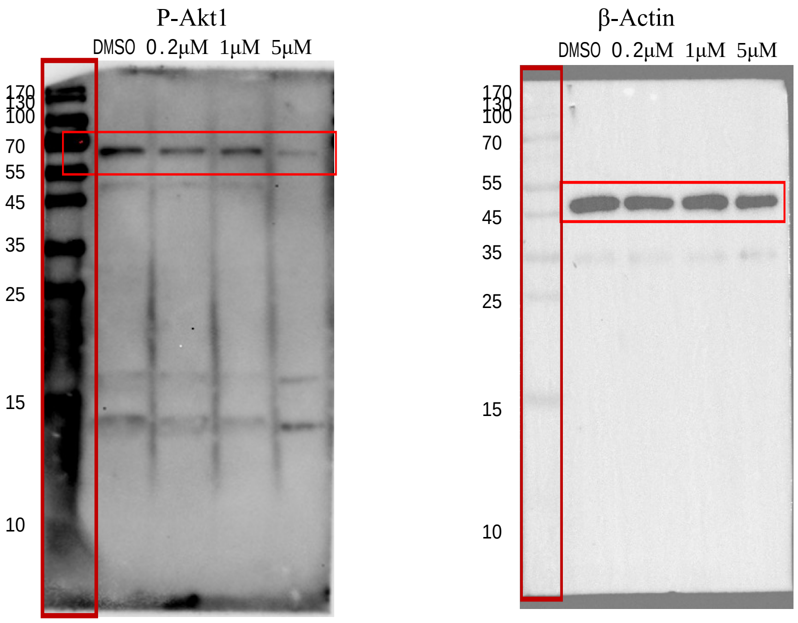

Fig. S7 Expression of Bcl-2 family proteins. (a), Western blotting analysis of Bcl-2 and Bcl- XL.

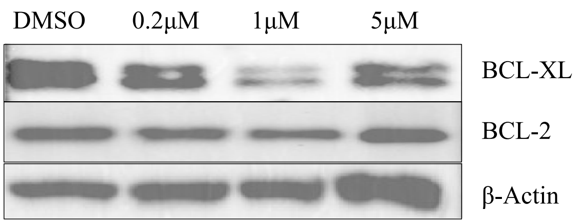

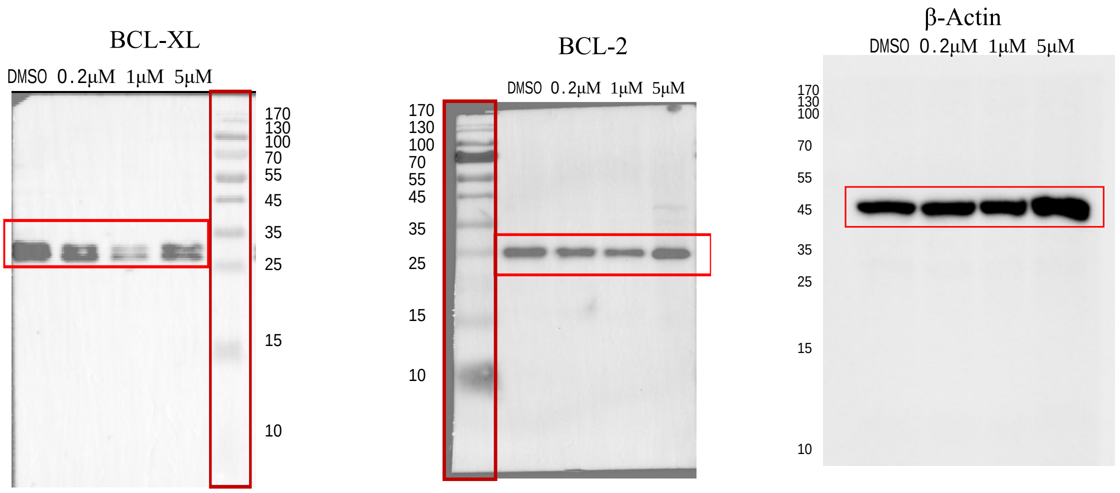

Fig. S7 Expression of Bcl-2 family proteins. (b), Western blotting analysis of BAX and BAD.

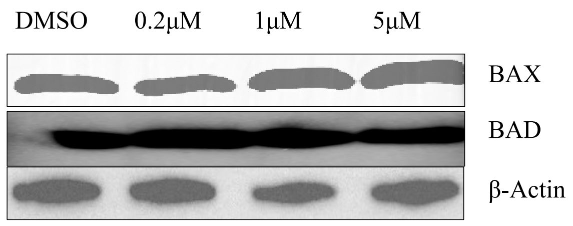

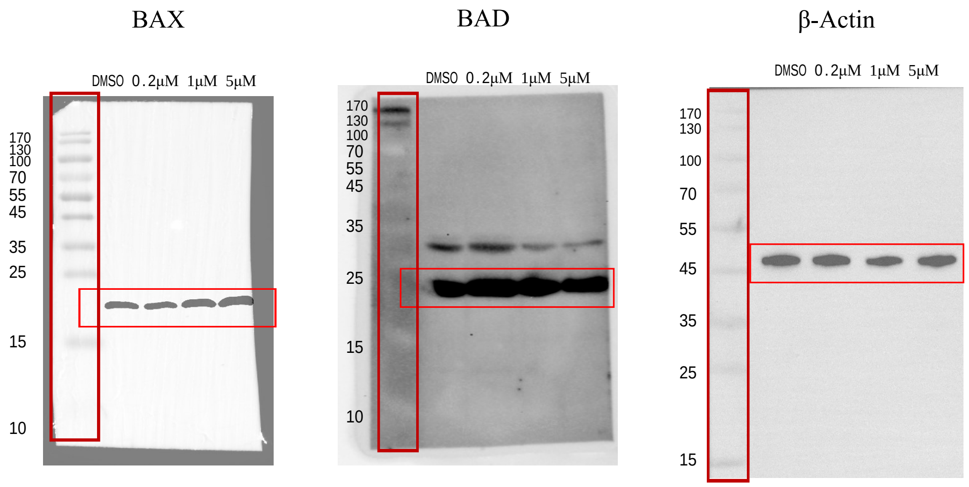

Fig. S8 Expression of Caspase family proteins. (**a**), Western blotting analysis of Caspase-3 and Cleaved-Caspase-3.

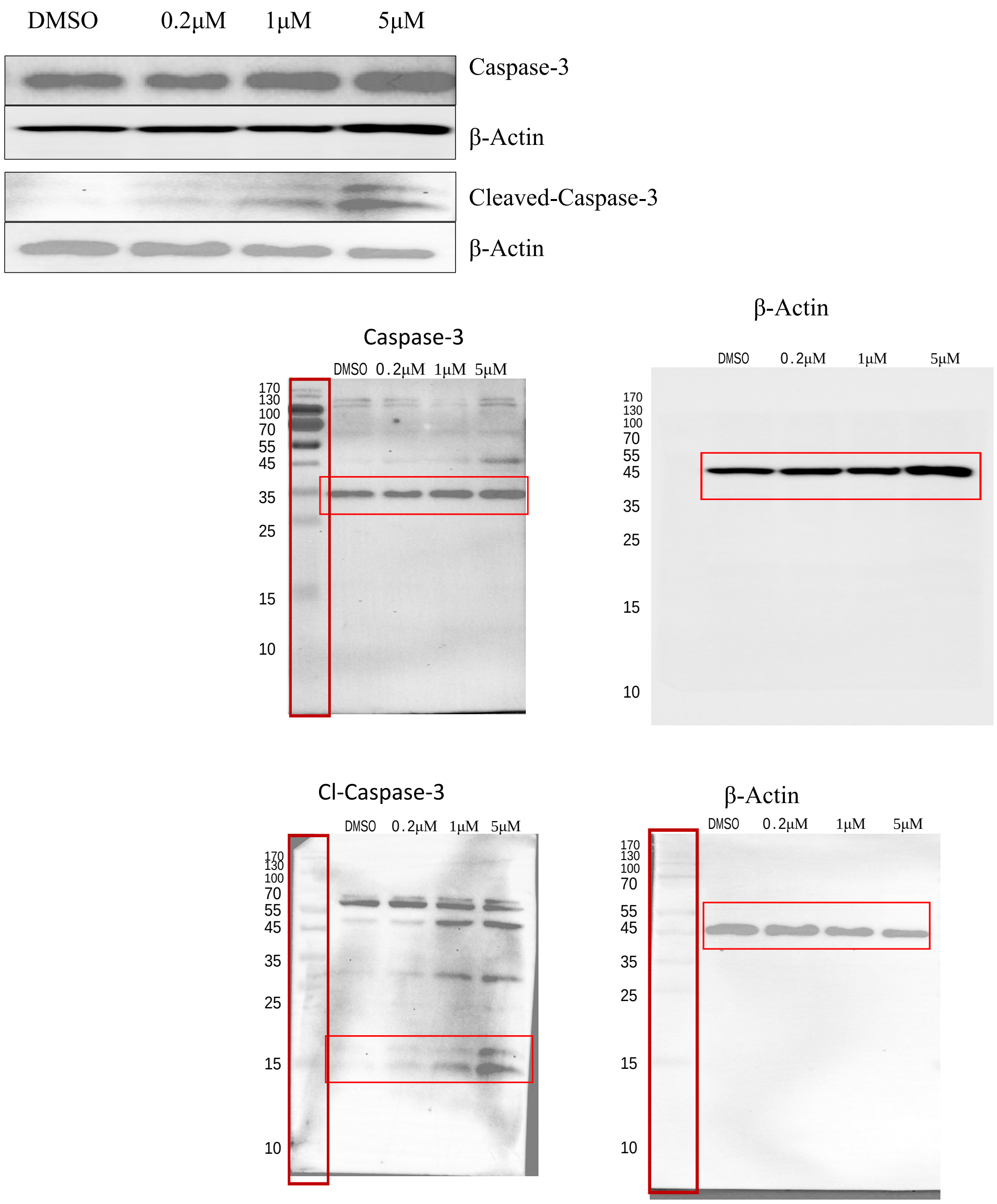

Fig. S8 Expression of Caspase family proteins. (**b**), Western blotting analysis of Caspase-8 and Cleaved-Caspase-8.

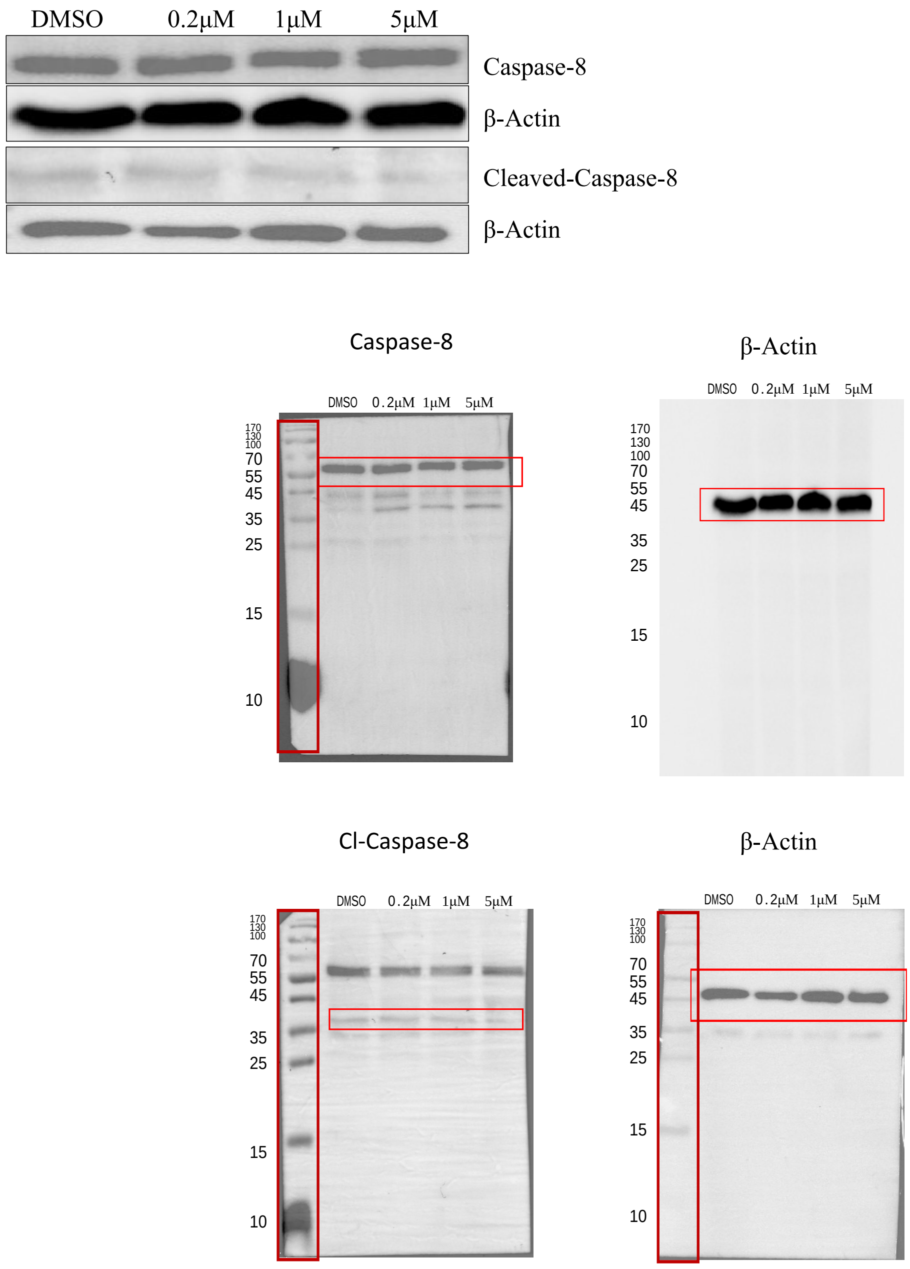

Fig. S8 Expression of Caspase family proteins. (**c**), Western blotting analysis of Caspase-9 and Cleaved-Caspase-9.

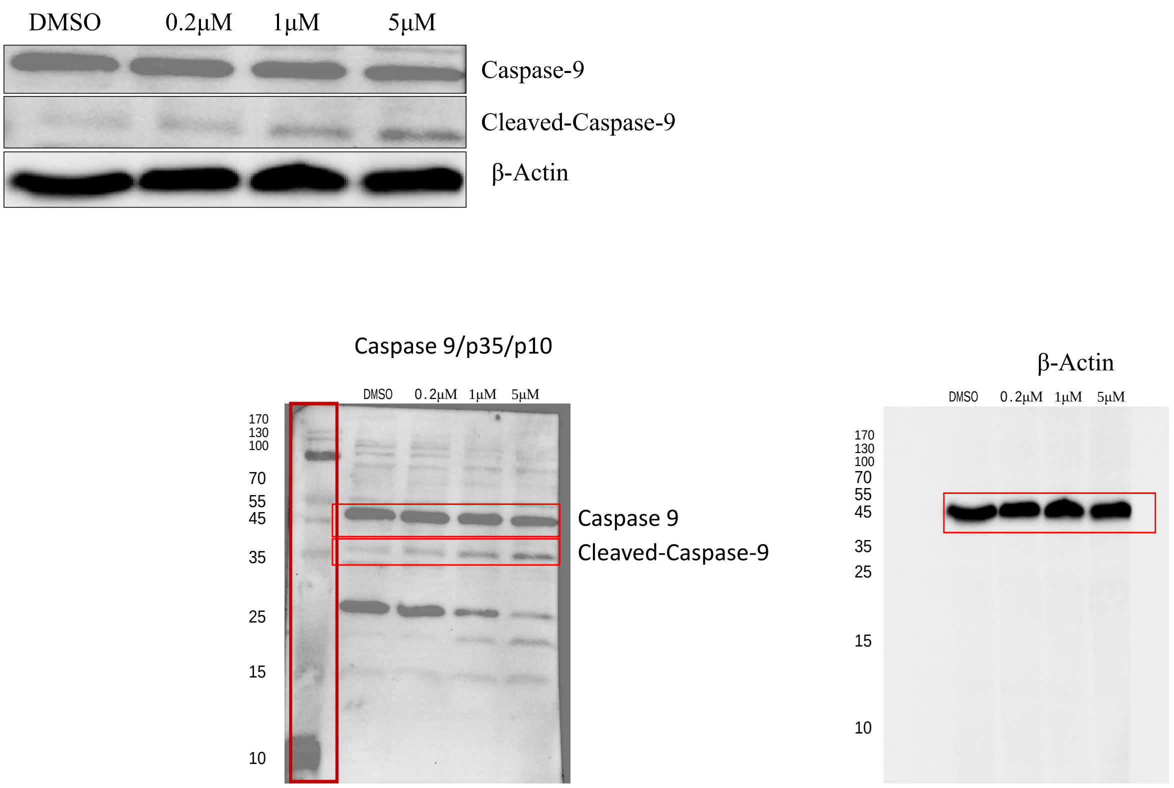

Fig. S9 Effect of NBU-2 on the induction of cell-cycle arrest. (b), Western blotting analysis of Cell cycle-related proteins. (CDK4, Cyclin 538 D1, p-Rb and p21).

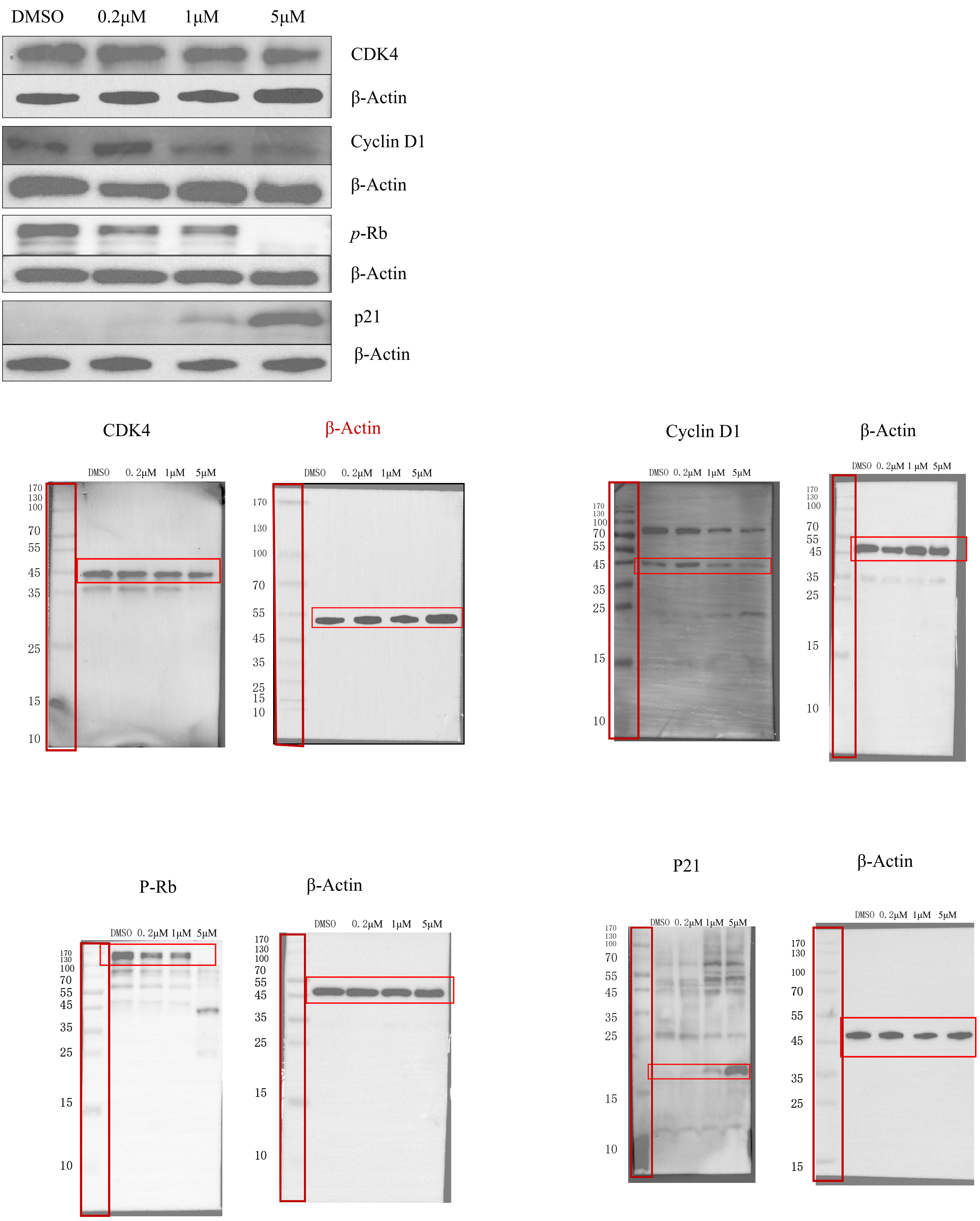

Supplement: Supplementary file 1 [file cells-14-01822-s001.zip › cells-3960132-supplementary/Supplementary Materials/Supplementary Material-Original Images for Blots.pdf]
