# Supplementary material for: Novel Nitrogen Heterocycle–Hydroxamic Acid Conjugates Demonstrating Potent Anti-Acute Lymphoblastic Leukemia Activity: Induction of Endogenous Apoptosis and G0/G1 Arrest via Regulation of Histone H3 Acetylation and AKT Phosphorylation in Jurkat Cells
Source: Cells. 2025 Nov 20;14(22):1822. doi: 10.3390/cells14221822 (PMC12651750; doi:10.3390/cells14221822)
Supplement: Supplementary file 1 [file cells-14-01822-s001.zip › cells-3960132-supplementary/Supplementary Materials/Supplementary Material.doc]

***Supplementary Material***

**Novel nitrogen heterocycle-hydroxamic acid conjugates demonstrating potent anti-acute lymphoblastic leukemia activity: Induction of endogenous apoptosis and G0/G1 arrest via regulation of histone H3 acetylation and AKT phosphorylation in Jurkat cells**

Lingjie Wu^1, #^, Li Zhao ^2, #^, Liping Wang ^1^, Yi Lu ^2^, Gaojie Lou ^2^, Bin Zhang ^2,^ * and Ning Wang ^1,^ *

^1^	School of Pharmacy, Health Science Center, Ningbo University, Ningbo, 315211, China; 2311390166@nbu.edu.cn (L.J.W.), 236004797@nbu.edu.cn (Y.L.), 236002485@nbu.edu.cn (G.J.L.), zhangbin1@nbu.edu.cn (B.Z.).

^2^ Institute of Drug Discovery Technology, Ningbo University, Ningbo, 315211, China; 2111072066@nbu.edu.cn (L.Z.), 2311130047@nbu.edu.cn (L.P.W.), wangning2@nbu.edu.cn (N.W.).

^#^ Authors with equal contributions.

***** Correspondence: zhangbin1@nbu.edu.cn; wangning2@nbu.edu.cn.

Table of Contents

1. Reagents	3

2. The structures and hydrogen atom numbers of intermediate **3**, **NBU-1** and **NBU-2**	5

3. Spectrograms of **NBU-1**	5

4. Spectrograms of **NBU-2**	7

5. WB raw data	9

# 1. Reagents

2-amino-3-methylbenzoic acid, 2,4-dichloronitrobenzene, palladium acetate, potassium phosphate, hydroxylamine hydrochloride, DPEPhos, sodium methoxide and Sodium tert-butoxide were purchased from Energy Chemical (Shanghai, China). Methyl 6-aminohexanoate hydrochloride was purchased from Bide Pharmatech Ltd. (Shanghai, China). K_2_CO_3_, NaOH, Et_3_N, DMF and NaCl were purchased from Shanghai Macklin Biochemical Co., Ltd. (Shanghai, China). H_2_SO_4_, HCl, and Cu were purchased from Sinopharm Chemical Reagent Co., Ltd. (Shanghai, China). 2-ethoxyethanol was purchased from Aladdin (Shanghai, China). Petroleum ether, ethyl acetate, methanol, ethanol, 1,4-dioxane and Dichloromethane were purchased from Shanghai Titan Technology Co., Ltd. (Shanghai, China). DMSO-*d_6_ and* Chloroform-*d* were purchased from Adamas-beta (Shanghai, China).

RPMI Medium Modified was purchased from Cytiva (Logan, UT, USA). Dulbecco's Modified Eagle Medium (DMEM) and penicillin-streptomycin were purchased from Gibco (Carlsbad, CA, USA). Fetal bovine serum was provided by pan-biotech (Adenbach, Germany). Trypsin-EDTA Solution with phenol red, annexin V-FITC apoptosis detection Kit, cell cycle and apoptosis analysis kit, cell counting kit-8 (CCK-8), reactive oxygen species assay kit, RIPA lysis buffer and NP-40 lysis buffer were from Beyotime (Shanghai, China).

Phosphate buffer saline(1×) (cell culture grade) PBS(1×) was purchased from Meilunbio (Dalian, China). ExpressCast PAGE Gel Preparation kit (7.5%, 10%, 12.5%,) and NcmECL Ultra Enhanced Chemiluminescent were from NCM Biotech (Suzhou, China). Tris(2-carboxyethyl)phosphine (TCEP) was purchased from MACKLIN (Shanghai, China). Iodoacetamide (IAA) and Microcon-30kDa Centrifugal Filter Unit with Ultracel-30 membrane (molecular weight cut-off, MWCO: 30 kDa, RC30 kDa) were provided by Sigma-Aldrich (St. Louis, MO, USA). Trypsin was provided by Promega(Madison, WI, USA).

Primary Antibodies: β-actin Mouse Monoclonal antibody, HDAC1 polyclonal antibody, HDAC6 polyclonal antibody, Bcl2 polyclonal antibody, Bcl-XL polyclonal antibody, P21 Polyclonal antibody, Caspase 8/p43/p18 polyclonal antibody, Caspase 9/p35/p10 polyclonal antibody and Caspase 3/p17/p19 polyclonal antibody were purchased from proteintech (Chicago, IL, USA). BAX rabbit polyclonal antibody, CDK4 Rabbit Polyclonal Antibody, Bad antibody, Phospho-Rb (Ser807) Rabbit monoclonal antibody, Histone H3 mouse monoclonal antibody, Acetyl-Histone H3 (Lys56) rabbit polyclonal antibody, Acetyl-Histone H4 (Lys5/8/12/16) mouse monoclonal antibody, Phospho-Akt1 (Ser473) rabbit monoclonal antibody, and Cyclin D1 rabbit polyclonal antibody were from Beyotime (Shanghai, China). Cleaved Caspase-3 (Asp175) (5A1E) rabbit mAb was from cellsignal (Danvers, Massachusetts, USA). HRP-conjugated Affinipure Goat Anti-Rabbit IgG(H+L) and HRP-conjugated Affinipure Goat Anti-Mouse IgG(H+L) were purchased from proteintech(Chicago, IL, USA).

# 2. The structures and hydrogen atom numbers of intermediate 3, NBU-1 and NBU-2

# 3. Spectrograms of NBU-1

^1^H spectrogram of **NBU-1**

^13^C spectrogram of **NBU-1**

HRESIMS spectrogram of **NBU-1**

# 4. Spectrograms of NBU-2

^1^H spectrogram of **NBU-2**

^13^C spectrogram of **NBU-2**

HRESIMS spectrogram of **NBU-2**

# 5. WB raw data

HDAC1

HDAC6

H3

Ac-H3

Ac-H4

*p*-Akt^(S473)^

BCL-2

BCL-XL

BAX

BAD

Caspase-3

Cl-Caspase-3

Caspase-8

Cl-Caspase-8

Caspase 9/p35/p10

Cdk4

P-Rb

P21

Cycline D1
